# The Impact of Three-Month Quercetin Intake on Quality of Life and Anxiety in Patients With Type II Diabetes Mellitus: An Early Data Analysis From a Randomized Controlled Trial

**DOI:** 10.7759/cureus.58219

**Published:** 2024-04-14

**Authors:** Aikaterini E Mantadaki, Manolis Linardakis, Marina Vafeiadi, Foteini Anastasiou, Aristidis Tsatsakis, Emmanouil K Symvoulakis

**Affiliations:** 1 Department of Social Medicine, University of Crete, School of Medicine, Heraklion, GRC; 2 Department of Social Medicine, Clinic of Social and Family Medicine, University of Crete, School of Medicine, Heraklion, GRC; 3 Department of Morphology, Laboratory of Toxicology, University of Crete, School of Medicine, Heraklion, GRC

**Keywords:** nutraceutical, phytochemical, type ii diabetes, anxiety, quality of life, diabetes mellitus, quercetin

## Abstract

Background: Diabetes is a high-prevalence, major chronic metabolic disease demanding effective interventions. Quercetin, a phytochemical with potential health benefits, has garnered interest for its therapeutic properties.

Aim: This study was designed to capture the early efficacy and clinical safety aspects following quercetin administration in patients with type II diabetes mellitus (T2DM).

Methods: The main study involved a randomized allocation procedure to assign non-insulin-treated patients attending the 4th Health Unit of Heraklion to intervention and control groups based on age and sex. The intervention group (n=50) received 500 mg of quercetin daily for 12 + (8 free intervals) + 12 weeks, alongside their usual treatment, while the control group (n=50) did not. After randomization, for the intermediary 12-week follow-up, data from 38 patients (intervention: 20; control: 18) were analyzed in this report. All subjects provided informed consent for the collection of anthropometric measurements, vital signs, daily habits data, and PiKo-6 spirometric readings. Additionally, participants responded to the Short Anxiety Screening Test (SAST) and the 36-Item Short Form Health Survey (SF-36) questionnaires.

Results: Thirty-eight participants were included (60% men and 40% women in the intervention group; 38.9% men and 61.1% women in the control group). In the treatment arm, Forced Expiratory Volume in the first second (FEV_1_) measured with PiKo-6 showed a *Δ*%- change for the intervention arm: +6.8%, control: -0.2% (p=0.059), systolic blood pressure; intervention: -7.4%, control: -3.7% (p=0.117), waist circumference; intervention: -1.5% control: -0.7% (p=0.455) and night-time sleep; intervention: +5.3%, control: +1.4% (p=0.926) were favourably influenced. The treatment group exhibited significant enhancements in both anxiety levels assessed by the anxiety symptoms scale (SAST-10, p=0.026) and quality of life evaluated by the SF-36 (p<0.001).

Conclusions: Positive evidence is emerging for a pleiotropic effect of quercetin intake in patients with T2DM, specifically in terms of anxiety reduction and amelioration of life quality, in just 12 weeks of administration and without adverse effects, indicating clinical safety and underscoring its potential for integration in T2DM supportive care.

## Introduction

Diabetes is a major chronic metabolic disease that affected 537 million adults worldwide in 2021, according to the International Diabetes Federation (IDF) Atlas, with a prediction of escalation to 643 million by 2030 and 783 million by 2045 [1]. Also, according to IDF estimates, the majority of nations spend between 5% and 20% of their overall healthcare budgets on diabetes, fact solidifying a challenging public health exigency label [2]. Diabetes is associated with increased production of free radicals, particularly due to the hyperglycaemia occurring in type II diabetes (T2DM) [3]. Moreover, the presence of pro-oxidants in the body disturbs the equilibrium between antioxidants and free radicals [4], in turn accelerating diabetes-related complications [5,6], including inflammaging [7].

Quercetin is a bioactive flavonoid that can be found in an abundance of plant foods, such as apples, onions, grapes and tea leaves [8-11] and has allured researchers. Two benzene rings connected by a pyran group with five free hydroxyl groups make up its fundamental structure [12]. In nature, it can be found as aglycones and glycosides, which are hydrolyzed in the intestinal lumen, whereupon they are absorbed in the form of quercetin aglycone by the human organism [13,14]. Almost 180 different quercetin glycosides have been described in nature [15], while the phytochemical is usually found at low levels, as a secondary metabolite of plants [16].

Quercetin shows significant antioxidant and anti-inflammatory capacity, which have been proven in vivo. Nevertheless, human and animal studies were ambiguous, perhaps due to different physiological processes commensurate with the species, as well as different levels of oxidative stress and inflammation [17]. Besides vasodilator, antithrombotic (anticoagulant) and cancer-preventing properties proven by in vivo studies [18-21], quercetin may also interfere with important cellular processes [22]. Furthermore, a study by Liu et al. (2006) indicated an improvement in the brain functions of D-galactose-induced aged mice associated with quercetin intake, with authors insinuating quercetin might also deliver natural anti-ageing properties [23]. Specifically, its antiaging effect has been studied exclusively in vitro, with promising results (rejuvenating effect) [24].

Quercetin has been identified as a Generally Recognized as Safe (GRAS) substance [25]. Notably, in a randomized controlled study (RCT) involving 1023 subjects aged 18-85 years who were administered either 500 or 1000 mg of quercetin daily for a period of 12 weeks, only 0.88% reported adverse effects and subsequently withdrew from the study [26]. Additionally, in a phase 1 dose-escalating trial where subjects infected with hepatitis C received higher quercetin doses ranging from 250 to 5.000 mg per day for 28 days, no adverse events or abnormal laboratory measurements were observed, indicating safety, tolerability and absence of toxicity within this cohort. The authors suggest that the maximum tolerated dose (MTD) is above 5.000 mg [27]. In fact, according to Umathe et al. (2009) who examined the effect of quercetin administration in mice with chemically induced diabetes due to alloxan, quercetin intake did not affect (non-statistically significantly) the bioavailability of per os administered pioglitazone [28]. Also, according to Kim et al. (2005), the intake of 500 mg of quercetin per day for 21 days did not affect the bioavailability of rosiglitazone in healthy subjects [29]. Numerous published human intervention studies including RCTs conclude that adverse effects concomitant with quercetin supplementation, including headache, stomach pain and numbness, have rarely been reported, were mild and associated with higher quercetin doses [16]. Yet, dedicated safety clinical trials in populations with T2DM are currently lacking.

Patients with diabetes often experience high levels of anxiety, negatively impacting their quality of life (QoL). This heightened anxiety adds to the existing challenges of managing their disease and may also play a role in the development of further complications, increasing the overall burden of the disease [30,31]. Patients with T2DM are often prescribed anti-anxiety medication to address their anxiety [32]. However, potential side effects associated with anxiolytics can sometimes discourage patients from continuing treatment, hindering effective diabetes management [33].

To carefully address the aforementioned safety gap and potential early signs of treatment efficacy, we performed an interim investigation regarding potential benefits resulting from quercetin supplementation in patients with T2DM, treated in usual care through a two-arm prospective randomized control trial in primary care (interventional vs observation group).

Despite the testified safety profile of quercetin and a conservative dosage, vigilance procedures were implemented, considering the clinical and nosological fragility of the participants. The study strictly adhered to the World Health Organization (WHO) and European Medicines Agency (EMA) Guidelines for good clinical practices for trials [34,35] and European Regulation 536/2014 [36].

Besides a diversity of factors following quercetin intake having been studied, to our knowledge, no other human study using an RCT scheme has examined its effect on anxiety, and few studies have addressed its impact on life quality [37,38]. Also, we are not cognizant of any other study encompassing a comparable breadth of factors related to quercetin supplementation as those explored within the context of this investigation.

## Materials and methods

Study design, population and setting

The study design was a two-arm, parallel prospective RCT, comprising an interventional vs control arm. The trial was prospectively registered at ISRCTN, trial registration number ISRCTN13131584, submission date 20-11-2022 (accessed on registry Jan 2024). This report summarizes the early results of the midway point (three-month quercetin supplementation period); regarding an ongoing 12+8+12 weeklong study that was granted permission by the Research Ethics Committee of the University of Crete (REC-UoC) (104/20-08-2021), also the 7th Health District of Heraklion, Greece (6380-14/02/2022), and has been responsibly registered and updated in the international ISRCTN database [39].

**Figure 1:**
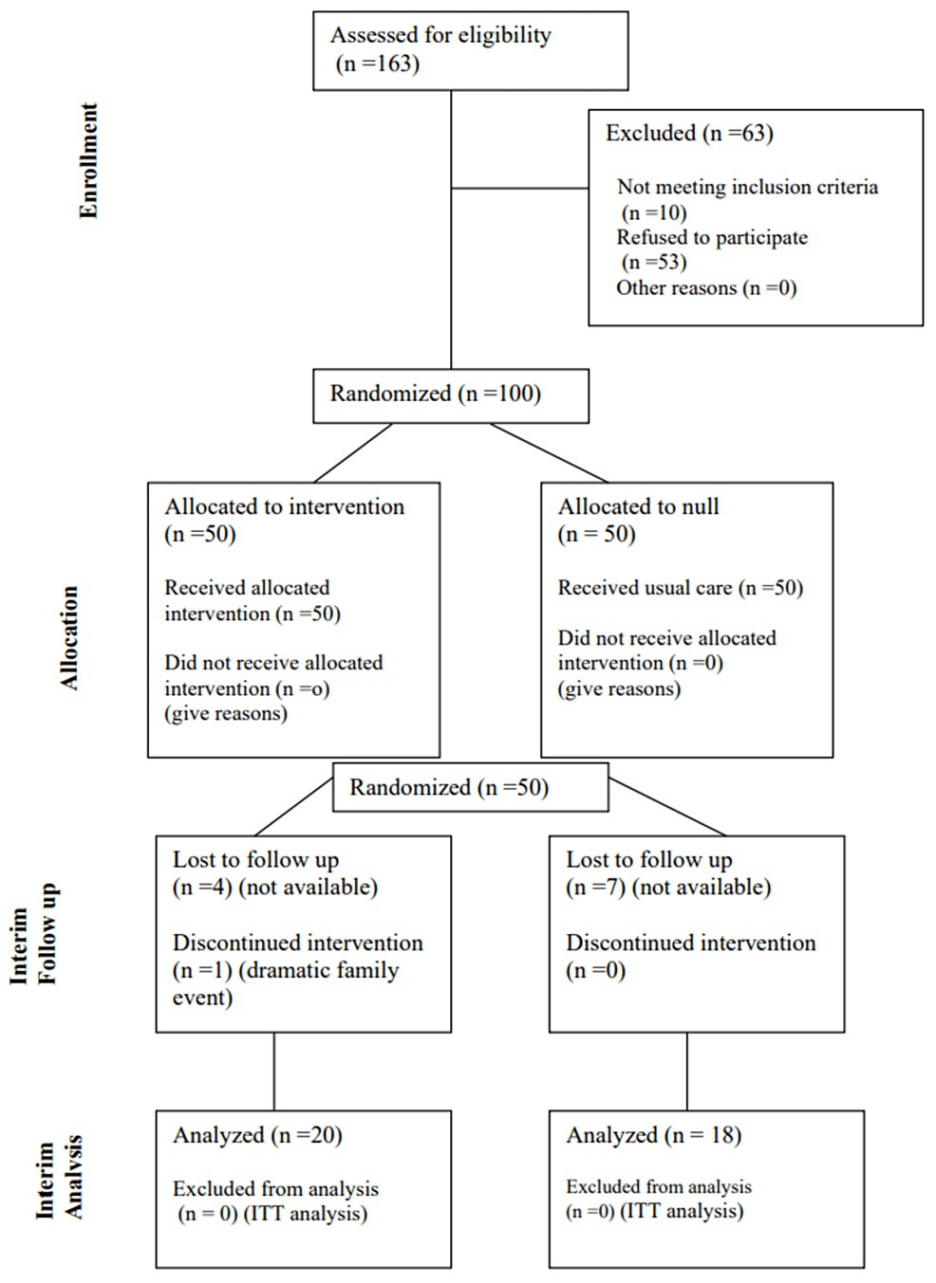
CONSORT trial flowchart for the needs of the three-month early data analysis. CONSORT: Consolidated Standards of Reporting Trials; ITT: intention to treat

All patients were recruited from the 4th Local Health Unit of Heraklion (4th TOMY), 7th Health District of Crete (Dec 2022-May 2023), whose medical and nursing staff provide primary health care services (PHC setting). They were receiving non-insulin medications for T2DM. An eligible patient pool was established by collecting and assessing demographic, anthropometric, health and lifestyle habits. Patients were allocated to the control and intervention groups using stratified randomization based on age and sex groups (1:1), as visualized in the flowchart designed to captivate the study’s course (Figure 1). Among 163 potential candidates, 10 did not meet inclusion criteria and 53 did not consent to participate. Therefore, a total of 100 patients were included.

An independent researcher supervised the random allocation sequence to prevent selection bias (allocation concealment). All patients were explicitly informed and complied to give written consent prior to participating in the study. Inclusion of patients was defined by age over 50 years, established diagnosis of T2DM confirmed by medical history and treatment with non-insulin medications as part of their standard care. The exclusion was granted for the most vulnerable patients, including cases of gestational diabetes (breastfeeding/pregnancy); maturity-onset diabetes of the young (MODY); pancreatogenic diabetes (T3cDM); heart, liver or kidney disease, serious psychiatric illness or addiction; patients with comorbidities such as cancer, AIDS, neoplastic diseases; organ transplant patients; patients under immunosuppressive therapy; patients who have recently suffered a cardiovascular event within three months before the start of the study; patients using other antioxidant supplementations; patients with a requirement for long-term use of aspirin, other than low dosage for protection against cardiovascular events, or non-steroidal anti-inflammatory drugs (NSAIDs); patients participating in another interventional clinical trial; patients with planned surgery or other interventional procedure requiring systemic anaesthesia during the study. The primary (anti-ageing effect) and secondary outcomes of the main study (biometric and clinical improvement) were elucidated in the study registration record [39]. The first participant was enrolled in February 2023. The randomized trial was conducted in compliance with the principles of the Helsinki Declaration to verify the safety of the patients [40]. A parallel group design was established and here we report respecting the CONSORT (Consolidated Standards of Reporting Trials) statement’s guidelines [41].

Intervention

Patients were randomly assigned to two groups: Control (CTR, n=50) and Intervention (INT, n=50). The CTR group did not receive any supplementary treatment other than their usual care (i.e. antidiabetic medications, excluding insulin). For the overall study duration, the INT group was supplemented for 12 + (8 free intervals) + 12 weeks with a single daily dose of 500 mg quercetin dihydrate formulation already circulating in the market and registered in the Greek National Organization for Medicines under disclosure no. 75608/20.11.2008, as an adjunct to their standard care.

The participants were assigned their intervention by referral to the allocation sequence, which was only disclosed to the principal investigator. Sealed containers of quercetin manufactured by Lamberts Healthcare LTD, each containing 60 tablets, with LOT number F160996, expiration date 03/2025, were supplied to the patients. The manufacturer formulates a quercetin content corresponding to the product’s specification (% of nominal content ± SD; 109.3 ± 9.3) as assessed by Vida et al. (2019) using high‐performance liquid chromatography (HPLC) [42]. The supplement ingredients were quercetin dihydrate, calcium carbonate, cellulose, anti-caking agents (stearic acid, silicon dioxide and magnesium stearate), tablet coating (hydroxypropyl methylcellulose, glycerin), crosslinked cellulose gum [43].

Also, a trained researcher offered standardized guidance to both CTR and INT groups during the study to maintain consistency and adhere to ethical principles.

All patients were frequently contacted by the appointed researcher in order to ensure prompt adherence to protocol requirements, via phone monitoring, which was enhanced by the provision of simple instructions, recording of tablets (pill counts) and kind reminders of treatment schedules. Additional reasons for not receiving treatment or not completing treatment or follow-up were listed.

Patients’ assessment

For the needs of the intermediate study, patients were interviewed at baseline (t=0) and after three months (t=12 weeks), while a full-variable assessment was scheduled for eight months. The three-month interim meetings were conducted during June through July 2023. The assigned researcher recorded health profile data by filling out a tailored to the study health information sheet, while depicting the biometric/clinical profile of patients. All patients underwent laboratory blood tests at baseline to estimate bio-clinical markers and were asked to answer the 36-item Short Form Health Survey (SF-36) [44-46] and Short Anxiety Screening Test (SAST-10) [47,48] questionnaires for the purposes of the study.

Measures

Height, weight, waist circumference and systolic (SBP) and diastolic blood pressures (DBP) of patients were measured using standard methods. We used the Tanita BC 730 weighing scale (TANITA, Tokyo, Japan, graduation 100g), the Seca 700 mechanical scale and the stadiometer (Medical Scales and Measuring Systems, Hamburg, Germany; precision of 0.1 cm, readability 50g) at baseline and at three months of intervention, tools utilized in an abundance of studies [49-54]. The Omron M2 basic automatic blood pressure monitor (Omron Healthcare Co. Ltd, Kyoto, Japan) was used for measuring blood pressure. Spirometry readings were performed using the PiKo-6 (nSpire Health GmbH, Oberthulba, Germany), in compliance with the recommendations given by the Lung Foundation of Australia [55]. PiKo-6, a proven-validity tool, is valuable for the evaluation of lung function, its measurements well correlate to those obtained by forced spirometry [56] and are considered to be superior in comparison to other microspirometres like COPD-6 [57]. Oxygen saturation was computed by pulse oximetry. Body mass index was calculated as weight (kg) divided by squared height (m^2^). The per cent value for *Δ*-change (*Δ*-change %) was calculated as the mean difference between pre- and post-supplementation values divided by the start values, multiplied by 100. Levels of complete blood count (CBC), C-reactive protein (CRP), haemoglobin A1C (HbA1c), reticulocytes, total cholesterol (TC), low-density lipoprotein cholesterol (LDL-C), triglycerides (TGL), creatinine, dehydroepiandrosterone sulphate (DHEAS), 25-OH-vitamin D and serum urea were measured at baseline using routine enzymatic assays in independent certified diagnostic laboratories.

The SAST-10 version used was validated and translated into the Greek language [47] and is considered a reliable screening tool in primary healthcare. It includes a 10-item Likert-type response scale and was first developed by Sinoff and colleagues for geriatric patients [48].

The SF-36 questionnaire was developed to survey health conditions [44] and is also a reliable measure of health-related QoL (Cronbach's alpha >0.85) [45]. We have utilized the Greek validated and normed SF-36 form, provided by the research team that has constructed it [46]. All questionnaires were filled out during the interview with the patients.

To mitigate biases stemming from patients' perceptions, standardized assessment tools with established validity and reliability were utilized. Supplementary strategies, including ensuring questionnaire items were clear and comprehensible by patients and administering them in a consistent and neutral manner, were implemented.

Statistical analysis

Statistical analyses were performed using the Statistical Package for the Social Sciences (SPSS) software (IBM Corp. Released 2019, IBM SPSS Statistics for Windows, v.25.0, Armonk, NY: IBM Corp.). Data were expressed with measures of central tendency and dispersion or frequency distributions as Δ-changes (%). Differences were explored by chi-square and Mann-Whitney tests. To compare levels and changes in health habits, body measurements, spirometry, blood pressure, QoL and anxiety between patients of INT and CTR groups, the Mann-Whitney test also was used. The critical value was set at 0.05.

## Results

Following an additional randomization protocol [58], 50 patients were randomly included in the preliminary part of this study, (INT: 25, CTR: 25). After communication with patients, 38 accepted the invitation to undergo the three-month investigation. Their data were analyzed in this first exploratory report, including 20 patients from the intervention group and 18 from the control group.

Compared with CTR, the INT group revealed no early statistically significant trend in the patients’ bioclinical profile, although descriptive albeit non-significant differences were identified in a favourable manner towards efficacy. Specifically, forced expiratory volume in the first second (FEV_1_) showed a *Δ*%- change of INT: +6.8%, CTR: -0.2% (p=0.059), result approaching statistical significance. Correspondingly, SBP INT: -7.4%, CTR: -3.7% (p=0.117) and waist circumference, INT: -1.5% CTR: -0.7% (p=0.455), night-time sleep INT: +5.3%, CTR: +1.4% (p=0.926) were found to be positively influenced. QoL and anxiety significantly improved in the INT group. Descriptive characteristics and laboratory measurements of the randomized patients included in the preliminary analysis at baseline are summarized in Tables 1-2 accordingly.

**Table 1:** Descriptive characteristics of 38 patients with type II diabetes mellitus (T2DM) randomized to intervention and control groups. Chi-square (χ^2^) and Mann-Whitney tests: * p<0.05; stand. dev.: standard deviation

				Groups	
				Intervention (n=20)	Control (n=18)
				n (%)	
Sex	Male			12 (60.0)	7 (38.9)
	Female			8 (40.0)	11 (61.1)
Age (mean±stand. dev.) (years)				65.9±9.1	66.6±7.7
Ethnicity	Greek			18 (90.0)	18 (100.0)
Current smoker			yes	5 (25.0)	6 (33.3)
Current drinker			yes	14 (70.0)	13 (72.2)
Multimorbidity		3+ morbidities		16 (80.0)	13 (72.2)
Polypharmacy		4+ medications		15 (75.0)	12 (66.7)
Vaccination		Influenza		16 (80.0)	15 (83.3)
		Pneumococcal		17 (85.0)	12 (66.7)
		Shingles (Herpes Zoster)		9 (45.0)	11 (61.1)
		COVID-19		19 (95.0)	18 (100.0)

**Table 2:** Levels of laboratory measurements at the beginning of the study of the 38 patients with T2DM randomized to intervention and control groups. Mann-Whitney tests; stand. dev.: standard deviation; LYMPH: lymphocytes; MONO: monocytes; NEUT: neutrophils; HGB: haemoglobin; PLT: platelet count; WBC: white blood cells; LDL-C: low-density lipoprotein cholesterol; CRP: C-reactive protein; HbA1c: Glycosylated hemoglobin; DHEAS: dehydroepiandrosterone-sulfate; T2DM: type II diabetes mellitus

	Groups		
	Intervention (n=20)	Control (n=18)	
	(mean±stand. dev.)		p-value
LYMPH (%)	29.6±5.5	30.6±7.6	0.988
MONO (%)	5.7±1.6	5.6±1.9	0.919
NEUT (%)	61.2±6.7	61.1±7.9	0.762
LYMPH x10^3^/μL	2.4±0.8	2.2±0.8	0.300
MONO x10^3^/μL	0.5±0.2	0.4±0.2	0.347
NEUT x10^3^/μL	5.2±2.2	4.7±1.6	0.773
HGB (g/dL)	14.2±1.1	13.4±1.3	0.017
PLT x10^3^/mm^3^	225.7±54.6	246.0±51.6	0.158
WBC x10^3^/μL	8.1±2.6	7.6±1.9	0.553
Reticulocytes %	1.4±0.6	1.1±0.3	0.080
Serum urea (mg/dL)	36.5±9.3	37.2±14.4	0.740
Blood creatinine (mg/dL)	0.8±0.3	0.9±0.3	0.784
Total cholesterol (mg/dL)	150.2±39.3	164.6±37.6	0.251
Triglycerides (mg/dL)	132.5±75.1	147.3±115.3	0.874
LDL-C (mg/dL)	78.0±30.5	83.9±21.7	0.390
CRP (mg/dL)	1.9±4.4	1.4±2.6	0.790
HbA1c (%)	7.1±1.2	7.0±1.4	0.784
25-hydroxy vitamin D (ng/mL)	33.9±15.1	36.3±18.6	0.784
DHEAS (μg/mL)	1.0±0.7	0.8±0.5	0.619
Cholesterol-ratio	3.8±2.3	3.6±1.2	0.874

Nineteen men (12 INT and 7 CTR) and 19 women (8 INT and 11 CTR) were included in this preliminary stage. Patients’ mean age was INT: 65.9±9.1 SD; CTR: 66.6±7.7. Current smokers were five in the INT and six in the CTR group and current drinkers were 14 in the INT and 13 in the CTR group. Multimorbidity, as defined in Table 1, was reported for 16 patients in the INT and 13 in the CTR group accordingly. Polypharmacy was registered in 15 subjects in the INT and 12 in the CTR group. Vaccination status was reported as follows: influenza (INT: 16; CTR: 15), pneumococcal (INT: 17; CTR 12), shingles (INT: 9; CTR: 11), COVID-19 (INT:19; CTR: 18). No patients were excluded during the three-month exploratory period, respecting the intention to treat (ITT) protocol and no adverse effects were reported. Blood test analysis revealed comparability of the baseline groups which was corroborated by the Mann-Whitney test (p>0.15), except for haemoglobin; the fact that can be easily explained due to a surplus of men in the INT and of women in the CTR group.

Effects of quercetin on health outcomes, anthropometry, spirometry, blood pressure, QoL and anxiety

**Figure 2:**
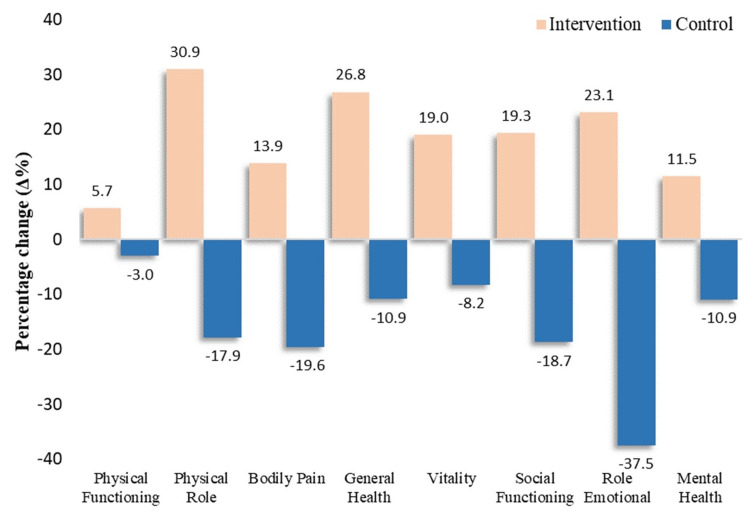
Percentage change in the eight components/subscales of the SF-36 Quality of Life Scale from baseline to three months of the study among the 38 patients with T2DM randomized to intervention and control groups. Significant changes in all components: Mann-Whitney tests in *Δ*-changes with p<0.05. SF-36: 36-Item Short Form Health Survey; T2DM: type II diabetes mellitus

Supplementation with quercetin for the first three months of the study was significantly correlated to a better overall QoL, regarding both the physical and mental component of SF-36 (p<0.001) and less anxiety (p=0.026) through lowering SAST-10 scores (*Δ*%- change INT: -5.1% vs CTR: +2.2%). More specifically, in the INT group, all eight components of the SF-36 (physical functioning, physical role, bodily pain, general health, vitality, social functioning, role emotional, mental health) QoL scale from baseline to three months of the study presented significant difference (Mann-Whitney tests in *Δ*-changes, p-value < 0.05). Table 3 provides the detailed results (including the summary components), further illustrated in Figure 2.

**Table 3:** Levels and changes of summary components and subscales of the SF-36 Quality of Life Scale from baseline to three months of the study among the 38 patients with T2DM randomized to intervention and control groups. Score 0-100 (higher indicates better quality of life); Mann-Whitney tests in *Δ*-changes; Cohen’s d was estimated as (mean Intervention-mean Control)/pooled stand. dev. stand. dev.: standard deviation; SF-36: 36-Item Short Form Health Survey; T2DM: type II diabetes mellitus

		Intervention (n=20)	Control (n=18)		
		(mean±stand. dev.)		p-value	Cohen's d
Physical Functioning	beginning	78.8±19.1	74.4±17.5		0.24
	3 months	83.3±17.1	72.2±21.2		0.25
	*Δ*-change	4.5±5.8	-2.2±8.4	0.003	0.25
Role Physical	beginning	68.8±46.5	77.8±42.8		0.39
	3 months	90.0±30.8	63.9±47.9		1.25
	*Δ*-change	21.3±40.8	-13.9±47.9	0.015	0.79
Bodily Pain	beginning	68.5±25.2	56.7±28.1		0.27
	3 months	78.0±20.9	45.6±32.0		1.33
	*Δ*-change	9.5±13.9	-11.1±27.6	0.001	1.03
General Health	beginning	56.0±22.4	53.6±24.2		0.10
	3 months	71.0±24.1	47.8±25.2		1.18
	*Δ*-change	15.0±13.6	-5.8±11.4	<0.001	0.50
Vitality	beginning	65.8±22.1	70.8±19.9		0.16
	3 months	78.3±19.1	65.0±21.3		0.49
	*Δ*-change	12.5±13.6	-5.8±15.6	<0.001	0.87
Social Functioning	beginning	55.0±45.6	63.2±44.0		0.19
	3 months	65.6±41.9	51.4±44.7		0.30
	*Δ*-change	10.6±30.7	-11.8±40.3	0.021	0.95
Role Emotional	beginning	65.0±48.9	88.9±32.3		1.00
	3 months	80.0±41.0	55.6±51.1		0.89
	*Δ*-change	15.0±36.6	-33.3±48.5	0.002	6.15
Mental Health	beginning	64.4±27.0	67.3±21.9		0.06
	3 months	71.8±24.6	60.0±21.6		0.50
	*Δ*-change	7.4±8.4	-7.3±14.1	<0.001	0.90
Physical Component	beginning	68.0±21.2	65.6±18.9		0.14
	3 months	80.6±19.5	57.4±22.8		0.63
	*Δ*-change	12.6±12.2	-8.3±15.8	<0.001	0.43
Mental Component	beginning	62.5±27.6	72.6±21.7		0.74
	3 months	73.9±22.9	58.0±30.2		0.92
	*Δ*-change	11.4±15.2	-14.6±22.9	<0.001	1.12

Changes were also observed in the INT vs CTR groups respectively. Night-time sleep (*Δ*%-change +5.3% vs +1.4%), moderate intensity activities in the last seven days (*Δ*%-change +62.1% vs +91.4%), waist circumference (*Δ*%- change -1.5% vs -0.7%), FEV_1_ (*Δ*%- change +6.8% vs -0.2%), forced expiratory volume in 6 seconds, FEV_6_ (*Δ*%- change +7.0% vs +3.1%), SBP, (*Δ*%- change -7.4% vs -3.7%), DBP, (*Δ*%- change -0.5% vs -0.4%), nonetheless not being significant. Table 4 shows favourably influenced trends observed in the INT vs CTR groups.

**Table 4:** Levels and changes of health habits, body measurements, spirometry, blood pressure, quality of life and anxiety of the 38 patients with T2DM randomized to intervention and control groups, from the beginning to three months of the study. Mann-Whitney tests in *Δ*-changes; ^a^ Score 0-100 (higher indicates better quality of life); ^b^ Score 10-40 (higher indicates higher anxiety); stand. dev.: standard deviation; FEV_1_: Forced expiratory volume in the first second; FEV_6_: Forced expiratory volume in 6 seconds; SF-36: 36-Item Short Form Health Survey; SAST-10: Short Anxiety Screening Test; T2DM: type II diabetes mellitus

		Groups		
		Intervention (n=20)	Control (n=18)	
		(mean±stand. dev.)		p-value
Night-time sleep (hours)	beginning	6.1±2.1	6.2±1.5	
	3 months	6.4±1.9	6.3±1.5	
	*Δ*-change	+0.3	+0.1	0.926
	*Δ*%-change	+5.3%	+1.4%	
Moderate intensity activities in the last seven days (days)	beginning	1.5±2.3	1.2±1.9	
	3 months	2.4±2.5	2.2±2.3	
	*Δ*- change	+0.9	+1.1	0.938
	*Δ*%- change	+62.1%	+91.4%	
Body Mass Index (kg/m^2^)	beginning	32.2±5.3	30.8±6.9	
	3 months	31.7±5.1	30.3±6.3	
	*Δ*- change	-0.5	-0.5	0.599
	*Δ*%- change	-1.4%	-1.5%	
Waist circumference (cm)	beginning	111.0±11.8	107.5±13.3	
	3 months	109.3±12.8	106.7±12.3	
	*Δ*- change	-1.7	-0.8	0.455
	*Δ*%- change	-1.5%	-0.7%	
FEV_1_ (L)	beginning	2.1±0.8	2.0±0.5	
	3 months	2.3±0.7	2.0±0.5	
	*Δ*- change	+0.1	0.0	0.059
	*Δ*%- change	+6.8%	-0.2%	
FEV_6_ (L)	beginning	2.5±1.0	2.3±0.6	
	3 months	2.7±0.9	2.4±0.6	
	*Δ*- change	+0.2	+0.1	0.188
	*Δ*%- change	+7.0%	+3.1%	
Systolic blood pressure (mmHg)	beginning	129.0±15.6	131.2±18.9	
	3 months	119.4±15.7	126.3±19.4	
	*Δ*- change	-9.6	-4.8	0.117
	*Δ*%- change	-7.4%	-3.7%	
Diastolic blood pressure (mmHg)	beginning	73.8±9.4	78.1±7.8	
	3 months	73.4±11.0	77.8±8.2	
	*Δ*- change	-0.4	-0.3	0.988
	*Δ*%- change	-0.5%	-0.4%	
Physical Component of Quality of Life (SF-36 Scale)	beginning	68.0±21.2	65.6±18.9	
	3 months	80.6±19.5	57.4±22.8	
	*Δ*- change	+12.6	-8.3	<0.001
	*Δ*%- change	+18.5%	-12.6%	
Mental component of Quality of Life (SF-36 Scale)^a^	beginning	62.5±27.6	72,6±21.7	
	3 months	73.9±22.9	58.0±30.2	
	*Δ*- change	+11.4	-14.6	<0.001
	*Δ*%- change	+18.2%	-20.1%	
Anxiety (SAST-10 Scale)^b^	beginning	21.4±2,3	22.6±3.8	
	3 months	20.3±1,9	23,1±2.6	
	*Δ*- change	-1.1	+0.5	0.026
	*Δ*%- change	-5.1%	+2.2%	

Qualitative information extracted by patients’ interviews (INT group)

Ameliorated sleep patterns were reported by two patients, lowered self-measured glucose levels and decreased arthritic pain by three, while eight have reported elevated energy levels. Two patients recommended the supplement to friends and family members with T2DM, due to an extremely positive perception.

Clinical safety and side effects

Besides available literature attesting to safety even in more intensive administration [27], no particular study has been conducted to examine safety within this nosologically burdened population. Thus, to ensure patients’ safety, vigilance procedures and practices were implemented. During the first three months of quercetin supplementation, no side effects were reported. One patient discontinued study participation due to a major family dramatic event.

Adherence to treatment

During the first three months concerning the initial phase of analysis, patients complied with intervention requirements. Reasons for postponed treatment initiation (less than one weeklong) or short-interval partial adherence comprised absent-mindedness, surgical procedures or concerns relevant to polypharmacy fear and potential cross-interactions, yet completion of the scheduled quantity intake was comparable within the treatment arm.

## Discussion

T2DM remains a global health challenge, with profound consequences for both physical and mental well-being. While existing therapies are essential, the search for safe and effective adjunctive strategies continues. Our three-month interim analysis investigated the effects of daily 500 mg quercetin (a flavonoid) as an adjunct supplement intake for patients already managing their T2DM. Results revealed significant improvements in mental and physical aspects of QoL (SF-36) and reduced anxiety (SAST scale) for treatment compared to the CTR group receiving standard-of-care alone. Another goal of this interim analysis was to monitor clinical safety in the absence of dedicated safety trials for this population. No side effects were reported. Additionally, we observed promising trends in spirometric capacity, SBP, waist circumference, and night-time sleep.

While quercetin's pleiotropic actions are widely discussed, relevant human studies remain limited. Our interim analysis results suggest quercetin intake may exert therapeutic benefits in reducing anxiety and improving QoL. This anxiolytic action is particularly significant for patients with T2DM, who often experience anxiety burdens negatively impacting self-management and disease complications.

Whereas the effects on vital signs, lung function, waist circumference, and sleep appear promising based on descriptive statistics, further investigation is needed to offer definitive conclusions, optimize treatment duration, dosage, and timing, examining better efficacy. More extensive studies are crucial to confirm these potential relationships and shed light on quercetin's full potential. Our initial findings are intriguing, particularly considering the lack of prior human research on quercetin's impact on anxiety reduction. This opens an exciting avenue for further exploration.

Recent research implies that flavonoids might have more complex protective mechanisms on cells than previously thought [59]. Specifically, their natural herbal form has limited absorption and gut bacteria can transform them into phenolic acids with potential health benefits still under investigation [60]. Interestingly, a study in mice showed that quercetin administered orally might have an anxiolytic effect as a prodrug, but becomes inactive after abdominal injection [61], indicating the route of administration may be essential. Additionally, the study hints at a possible link between the number of hydroxyl groups in a specific ring of the flavonoid molecule regarding its effect. Furthermore, its conversion to the metabolite 4-hydroxyphenylacetic acid appears to have greater antiplatelet activity than quercetin itself [62]. This in vivo study proposes that quercetin's anxiety-reducing effects may depend on gut microflora. Of note, antibiotic-induced sterilization led to a loss of quercetin's anxiolytic activity, even when administered orally. This highlights the pivotal role gut bacteria may play in metabolizing quercetin into a bioactive form [62].

Supporting our findings, previous mice-model studies have demonstrated quercetin's beneficial role in anxiety regulation, attributed to the reduction of oxidative stress in the hippocampus [63-66]. Our study is the first to explore this effect in humans.

Furthermore, ageing has been associated with decreased QoL, particularly in individuals over 80 years old [67]. Early research indicates a connection between anxiety disorders and accelerated signs of neuroprogression in ageing [68]. Elevated anxiety levels have also been linked to lower QoL in seniors [68]. Additionally, ageing anxiety has been identified as a predictor of death anxiety, further contributing to a lower QoL [69].

Our study highlights the importance of QoL improvements in diabetes management. QoL, which includes physical, emotional, and social well-being, is often negatively impacted in individuals with T2DM [70]. While complete remission is not yet available, preventing further QoL decline is essential to optimal diabetes care [71]. This focus goes beyond traditional clinical measures centred solely on disease control.

An RCT by Dehghani et al. (2021) investigated quercetin's effect in post-myocardial infarction patients and observed an improvement in QoL, specifically in the dimension of insecurity [37]. However, to our knowledge, no other aspects of QoL were found to be influenced by quercetin intake. Our results demonstrate a broader improvement in QoL. While not directly comparable due to different populations, our findings hinting at quercetin's potential benefits for QoL may exceed those reported in a two-month, placebo-controlled clinical trial involving 500 mg per day of quercetin supplementation among chronically fatigued (*Δ*%- change, p; + 3.131%, 0.992) [38].

Moreover, our preliminary analysis revealed a detectable, though not statistically significant, improvement in lung function (*Δ*-change in FEV_1_; INT: +0.1, CTR: 0.0, p<0.059). This may be related to the potential strengthening of the diaphragmatic muscles, an intriguing possibility that warrants further exploration. Indeed, research has shown a link between oxidative stress mechanisms and compromised diaphragmatic muscle efficiency in patients with severe chronic obstructive pulmonary disease (COPD) [72]. Notably, our findings align with previous animal studies demonstrating quercetin's potential to enhance pulmonary function, comprising a study in male mice [73] and research showing protection against hyperoxic pulmonary damage in newborn mice [74]. More recently, a small double-blind, placebo-controlled clinical trial involving 14 COPD patients advocates that quercetin might effectively reduce inflammation in the lungs [75]. The authors propose that this may lead to improved lung function by reducing the influx of inflammatory cells (macrophages, lymphocytes, and neutrophils), downregulating pro-inflammatory cytokine release (IL-10, IL-13, and IL-22), decreasing oxidative stress markers, increasing antioxidant enzyme activity, protecting against excessive extracellular matrix degradation by enzymes (MMP9 and MMP12) and indirectly enhancing Nrf2-driven antioxidant defences [73].

Regarding blood pressure, our study detected descriptive improvements without statistical significance. Similarly, an RCT by Zahedi et al. found no significant difference in SBP between quercetin and placebo groups in women with T2DM [17]. Conversely, a systematic review and meta-analysis by Serban et al. reported significant decreases in blood pressure after quercetin supplementation [76]. They imply that quercetin's antihypertensive effects are not fully elucidated and may arise through diverse mechanisms, including reducing oxidative stress, improving endothelial function, and influencing cell signalling pathways and gene expression [76].

Importantly, our patients reported improved sleep cycles. This is noteworthy as research contends persistent sleeplessness may accelerate cellular ageing [77]. Additionally, a murine study indicates that quercetin might influence sleep patterns by activating GABA(A) receptors [78], a possibility supported by some of our patients' experiences. However, an RCT including 58 men and women in the Reserve Officers' Training Corps (ROTC) showed no impact of quercetin intake on vitality, exhaustion, or sleep quality [79]. Remarkably, our results show effects similar to those of *Bacopa monnieri* extract on sleep quality in sleep-deprived adults [80] and of *Mentha pulegium* extract on QoL of patients with functional dyspepsia [81].

We also observed a non-significant decreasing trend in waist circumference in our INT group. This aligns with a systematic review by Huang et al. (2019) in which quercetin intake did not significantly alter waist circumference [82].

While our patients reported noticeable blood glucose improvements, a prior study involving T2DM patients showed quercetin did not affect glycemic regulation, which could be due to the lower dosage (250 mg) and different baseline characteristics compared to our study [83]. Finally, an original research study showed that daily consumption of 500mg quercetin could alleviate rheumatoid arthritis symptoms, an observation also supported by our patients [84].

Summarizing, one could hypothesize quercetin supplementation may contribute to lowering cardiometabolic burden [85,86], by synergistically mitigating blood pressure, abdominal obesity, and anxiety [87]; improving lung function [88-90] and prolonging sleep time [91]. Yet, hitherto, since this study focused specifically on patients with T2DM, only wider analysis could provide more robust statistical evidence and determine the generalizability of these findings to other populations. The lack of significant effects of quercetin supplementation might be related to a conservative selection of administration dosage within the recommended range, affected by the length of the study, considering an intermediate investigation and needs to be explored by further relevant studies. Still, our main research result expectations have been enhanced after our interim data interpretation.

In conclusion, our study's focus on a PHC setting underscores quercetin's potential for integration into T2DM research. The observed positive trends in anxiety reduction, QoL improvement, sleep, blood pressure, and waist circumference suggest a possible holistic, patient-centred supportive care within the PHC context. As a natural alternative, quercetin could potentially reduce reliance on traditional anxiolytics, mitigating side effects for patients with T2DM.

These promising interim results from our ongoing RCT warrant further investigation to definitively establish quercetin's long-term efficacy and safety. Larger-scale clinical trials are critical to determine optimal dosage regimens, elucidate mechanisms of action, and gather robust evidence supporting its broader implementation as a complementary or alternative therapy within comprehensive diabetes care plans in primary care.

Study strengths and limitations

To begin with, the study had a parallel, two-arm design and was conducted as a non-blinded trial, thus raising the risk of performance bias. Additionally, subsidiary inaccuracies due to a possible ‘active treatment response’ cannot be ruled out.

In fact, in this report, we provide only clinical and health profile data, without any attempt to unveil underlying mechanisms of modulation, elucidating the properties of quercetin supplementation.

The present study is the first study exploring quercetin's potential pleiotropic benefits through an RCT design encompassing an amalgam of multiple exploration variables, in a simultaneous manner. Randomization was successful in generating comparable baseline groups with respect to multimorbidity prevalence, demographic, anthropometric and laboratory data. Nonetheless, there are certain limitations that need to be given consideration when assessing the current observations from the process. It was noted that patients belonging to the CTR group were generally less motivated to set up a meeting and less interested in participating in the intermediate three-month encounter. The ‘no-treatment’ option can be seen as a reason for major attention for the final eight-month recruitment call, since a CTR participation without a placebo preparation intake, beyond the blindness lack, may lead to feelings of passive interaction.

Also, despite its random allocation, our sample is limited in terms of size and one-setting participation. Larger studies and multi-setting designs may offer more robustness to the already emerging findings, regardless of the acknowledged limitations.

Additionally, the study was targeted at patients with T2DM, due to the premature ageing burden [85] and thus, benefits from quercetin intake might not be fully tangible, as in the case of healthier population groups of the same age.

The outcomes of this preliminary analysis were based on collected health information and perceptions, without ignoring a potential source of bias. However, the use of internationally validated scales on QoL and anxiety may buffer this limitation.

## Conclusions

The first results obtained from this study are promising and positively indicate quercetin supplementation is clinically safe and well tolerated. Even in a vulnerable, high-morbidity population group with diabetes, some benefits are strongly presumed through favourable trends captured by descriptive comparisons and fewer significant results in the first three months of intake. Our results provide insights regarding the efficacy of a nutraceutical, quercetin, in terms of enhancement of daily health aspects and advanced knowledge in the realm of QoL amelioration and anxiety decrease, underscoring its potential for integration into T2DM supportive care. The observed positive trends suggest a possible holistic, patient-centred approach within the PHC context. In general, our preliminary investigation yields some input in the field of phytochemical supplements and evokes the need for further research. This preliminary analysis illustrates the efficacy, clinical safety, tolerability and feasibility of this intervention in a primary care environment.
